# Influence of Intraoperative Fluid Management on Postoperative Outcome and Mortality of Cytoreductive Surgery for Advanced Ovarian Cancer—A Retrospective Observational Study

**DOI:** 10.3390/healthcare12121218

**Published:** 2024-06-19

**Authors:** Claudia Neumann, Eva Kranenberg, Alina Schenk, Nicholas Kiefer, Tobias Hilbert, Sven Klaschik, Mignon Denise Keyver-Paik, Martin Soehle

**Affiliations:** 1 Department of Anaesthesiology and Intensive Care Medicine, University Hospital Bonn, 53127 Bonn, Germany; eva.kranenberg@ukbonn.de (E.K.);; 2 Institute of Medical Biometry, Informatics and Epidemiology, University Hospital Bonn, 53127 Bonn, Germany; 3 Association of Catholic Clinics of the City of Düsseldorf, 40479 Düsseldorf, Germany; 4 Women’s Clinic, Medical Campus Wolfsburg, 38440 Wolfsburg, Germany

**Keywords:** fluid management, cytoreductive surgery, ovarian cancer

## Abstract

**Background:** The surgical treatment of advanced ovarian cancer is associated with extensive tissue trauma, prolonged operating times and a considerable volume shift. It, therefore, represents a challenge for anaesthesiological management. **Aim:** The aim of this single-centre, retrospective, observational study was to investigate whether intraoperative extensive volume supply influences postoperative outcomes and long-term survival. **Methods:** The study included 73 patients with a mean (SD) age of 63 (13) years who underwent extensive tumour-reducing surgery for ovarian cancer between 2012 and 2015. The effect of the intraoperative fluid balance on postoperative complications, such as anastomotic insufficiency or pleural effusions, was investigated using logistic regression. Further, the influence of fluid balance, lactate and creatinine levels on 5-year survival was analysed in a Cox regression model. Associations between anaesthesia time and the intraoperative fluid balance were examined using Spearman’s rank correlation coefficients. **Results:** The mean (SD) postoperative fluid balance in the considered patient cohort was 9.1 (3.4) litres (l) at a mean (SD) anaesthesia time of 529 (106) minutes. Cox regression did not reveal a statistically significant effect of the fluid balance, but it did reveal a statistically significant association between the lactate level 24 h following surgery and the 5-year survival (HR [95%-CI] fluid balance: 0.97 [0.85, 1.11]; HR [95%-CI] lactate: 1.79 [1.24, 2.58]). According to logistic regression, the intraoperative fluid balance was associated with an increased chance of postoperative complications in the considered patient cohort (OR [95%-CI] 1.28 [1.1, 1.54]). **Conclusions:** We could not detect a negative impact of an increased fluid balance on 5-year survival, but a negative impact on postoperative complications was found in our patient cohort.

## 1. Introduction

### 1.1. Background/Rationale

Epithelial ovarian cancer represents the most lethal gynaecologic malignancy. In Germany, ovarian cancer accounts for one-third of all malignant neoplasms of the female genitals with an incidence of 7000 cases per year [1]. So far, optimal cytoreduction is the only prognostic factor which may be positively influenced by a skilled ovarian cancer team [2]. In order to reach this goal, surgical complexity has increased over the last three to four decades tremendously [3,4]. Despite all surgical efforts, the 5-year survival rate is still around 42% [5].

This is mainly explained by the fact that the tumour is often discovered in late stages, and there is still no successful strategy for early detection.

According to current data, extensive surgical cytoreductive therapy is a promising approach, especially for the higher-grade tumour stages of categories FIGO III and VI [6]. Depending on the extent, histology and hormone receptor status of the tumour, radio- and/or chemotherapy is usually administered additionally to improve the chances of survival [7]. Extensive cytoreductive surgery is challenging not only for the gynaecologist but also for the anaesthetist. Depending on the tumour extent and the involvement of other organs, procedure times are long, there is extensive tissue trauma, the action of peritoneal stripping and, subsequently, considerable fluid shifts. An inflammatory reaction that already starts intraoperatively can also result in the necessity of infusing further fluids and the additional use of catecholamines to maintain a stable circulatory situation [8].

Extensive intraoperative volume administration is critical, as it may be associated with an increased risk of postoperative complications [9,10]. On the other hand, restrictive management always harbours the risk of hypovolemia with further end-organ deficiency. Nevertheless, in order to optimise oxygen delivery to avoid tissue hypoxemia, it is important to ensure a sufficient volume status to ensure adequate cardiac output (CO) [11].

Restrictive fluid management has been recommended by some clinicians, but no evidence of a survival benefit for patients with restrictive volume management has been reported so far [12].

### 1.2. Objectives

The aim of this study was to investigate the influence of intraoperative fluid administration regarding postoperative complications, as well as the short- and long-term survival, of patients undergoing cytoreductive surgery for advanced epithelial ovarian cancer.

The secondary outcomes included the correlation of the amount of fluid infused with the surgical incision–suture time, anaesthesia time, ventilation time and catecholamine requirements. Postoperative lactate and creatinine levels were recorded to assess the impact of fluid volume on tissue oxygenation and renal function.

## 2. Methods

### 2.1. Study Design

With approval from the local ethics committee, patients who required extensive cytoreductive surgery due to the diagnosis of advanced ovarian cancer in the period from January 2012 to December 2015 were included in this retrospective, observational, single-centre study. Patients were excluded if their anaesthesia record was unavailable or if a different tumour histology was diagnosed by the pathologist afterwards. Due to the retrospective character of the study, informed patient consent was waived.

### 2.2. Variables

Demographic data on patients’ age (in years), height (cm) and weight (kg) were collected. All patients received general anaesthesia with mechanical ventilation using propofol, cisatracurium, isoflurane and sufentanil or remifentanil. The anaesthetist decided whether to employ volume or catecholamines in order to maintain a mean arterial pressure of 60 mmHg, in accordance with the hospital standard.

Intraoperative monitoring included an invasive arterial line and a central venous catheter. The recorded parameters comprised heart rate, blood pressure, oxygen saturation, blood gas analyses, fluid administration, and the administration of blood products and medication.

Fluid intake was calculated as the sum of infused crystalloid and colloid solutions, as well as of blood products such as red blood cell concentrates (290 mL/unit), fresh frozen plasma (290 mL/unit) and platelet concentrates. Fluid outtake was assessed by summing the urine output and blood loss. Fluid balance was defined as the fluid intake minus the fluid outtake.

In addition, the anaesthesia time and duration of surgery in minutes were recorded. The postoperative transfer to the intensive care unit (ICU) and the length of the ICU stay (in minutes) were assessed, as was the occurrence of postoperative complications. In detail, the following complications were recorded:

- Anastomotic insufficiency;
- Pleural effusion;
- The need for postoperative ventilation.

The level of serum lactate (mmol/L) at the end of surgery and 24 h following surgery was used to assess tissue trauma, and with regard to volume management, the creatinine value (mg/dL) on postoperative days 1 to 3 was determined to assess a possible influence of the volume regime on renal function. Finally, the mortality rate 1 year after surgery was assessed, together with a long-term follow-up after 5 years. The review of long-term survival was conducted as part of the documentation for the gynaecologists’ cancer registry and was last updated in January 2022.

### 2.3. Data Sources/Measurement

Vital signs, as well as the administered drugs, fluids and blood products, were recorded and evaluated using paper protocols. In addition, patient data such as laboratory values, documented complications and survival status were recorded from electronic patient files.

### 2.4. Bias

Due to the different extensiveness of the surgical procedures, the patient collective was rather heterogeneous. Nevertheless, this observational study aimed to provide an overview of the outcomes for patients with overall advanced ovarian tumour stages and shed light on the intra- and postoperative challenges, complications and survival of patients with this type of cancer.

### 2.5. Statistical Methods

Statistical analysis was performed using R and the environment for statistical computing (version 4.3.2). Continuous variables are reported as means (SDs) or median [IQR], as indicated. Categorical variables are reported as absolute and relative frequencies. Spearman’s rank correlation coefficient with a 95% confidence interval was employed to assess the correlations between fluid intake/balance and various parameters, including the surgical incision-to-suture time, the anaesthesia time, the ventilation time, the maximum amount of noradrenaline, lactate levels (24 h postoperatively) and creatinine. The impact of the fluid balance on the occurrence of complications, comprising any complication, pleural effusion and anastomotic insufficiency leaks, was examined through logistic regression, incorporating odds ratios with 95% confidence intervals (OR [95%-CI]).

To evaluate overall survival, the time from the end of surgery to death from any cause was measured in days. Patients were considered censored at 5 years if the event of interest had not occurred within 5 years following surgery or at the time the patient was lost to follow-up.

Unadjusted 1-year and 5-year survival was evaluated using the Kaplan–Meier estimate with 95% confidence intervals. The effect of the fluid balance, the lactate level (24 h postoperatively), the occurrence of any complication, the surgical incision-to-suture time, age at the baseline and the maximum amount of noradrenaline on overall 5-year survival was analysed using a Cox regression model. For the Cox regression model, hazard ratios (HRs) with 95% confidence intervals are reported. The significance level was set to 5%, and *p*-values below 5% were considered being statistically significant.

## 3. Results

In total, the study cohort comprised 73 female patients characterised by a mean age of 63 (13) years, an average height of 165 (6) cm and a mean weight of 71 (16) kg. Additional, detailed patient characteristics are provided in Table 1.

**Figure 1:**
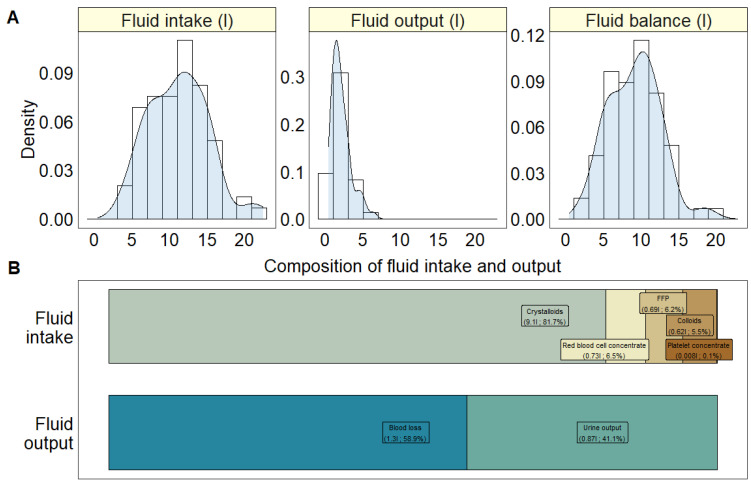
(**A**) Histograms of fluid intake (L), fluid output (L) and fluid balance (L) in the patient cohort. (**B**) Visualisation of the average contribution of red blood cell concentrate, FFP, platelet concentrate, colloids and crystalloids to the total fluid intake and the average contribution of urine output and blood loss to the fluid output.

The total fluid intake is composed of the quantities of red blood cell concentrate, FFP, platelet concentrate, colloids and crystalloids, each measured in litres (Table 2). Crystalloids, on average, made the most substantial contribution, accounting for 9.1 L or 81.7%, as illustrated in Figure 1B. In contrast, the total fluid output was calculated according to the blood loss and urine output, with a mean contribution of 1.3 L (58.9%) and 0.87 L (41.1%), respectively. The fluid balance was defined as the total fluid intake minus the total fluid output for each patient.

**Figure 2:**
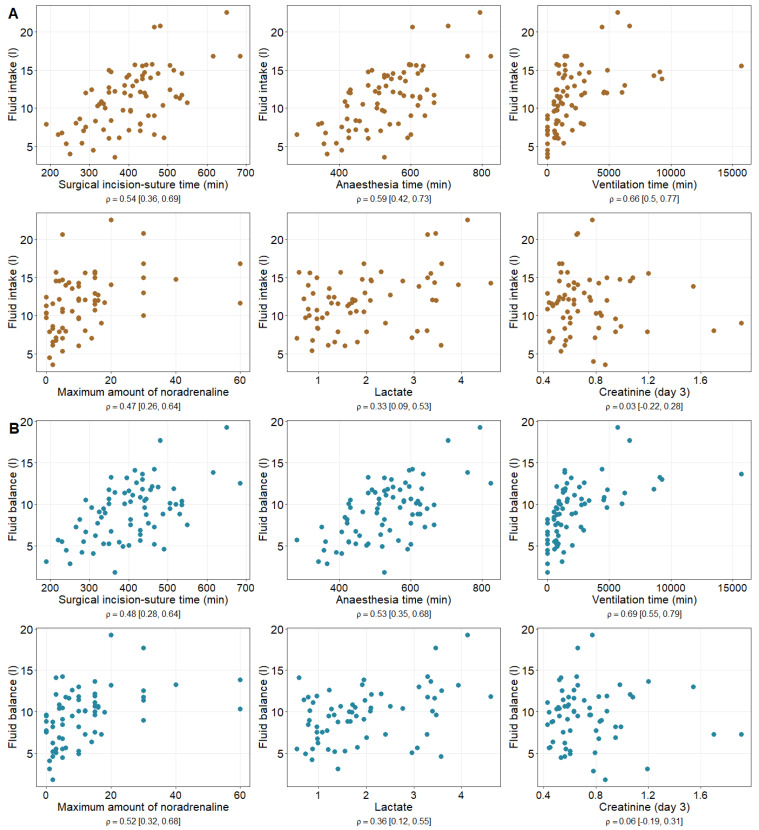
Visualisation of correlation between fluid intake (**A**) (fluid balance (**B**)) and anaesthesia, surgery times and laboratory measures.

The surgical incision-to-suture time, anaesthesia time, as well as ventilation time, were significantly positively correlated with both fluid intake and fluid balance (Table 3 and Figure 2). We found a significantly positive correlation between the maximum amount of noradrenaline and fluid intake (fluid balance), as well as between the lactate level 24 h postoperatively and the fluid intake (fluid balance). We did not find a significant correlation among the fluid intake or fluid balance and the creatinine level at day 3 postoperatively (Table 3 and Figure 2).

Logistic regression models revealed a statistically significant influence of the fluid balance on the occurrence of any complication (OR 1.28 [1.1, 1.54]), the presence of pleural effusion (OR 1.33 [1.12, 1.63]) and the occurrence of anastomotic leaks (OR 1.45 [1.12, 2.05]). It should be mentioned here that no differentiation was made between the surgical placement of one or multiple anastomoses in the case of anastomotic insufficiencies. The provided ORs are based on a one-litre increase in the fluid balance. Consequently, higher fluid balance values were associated with an elevated chance of developing complications.

**Figure 3:**
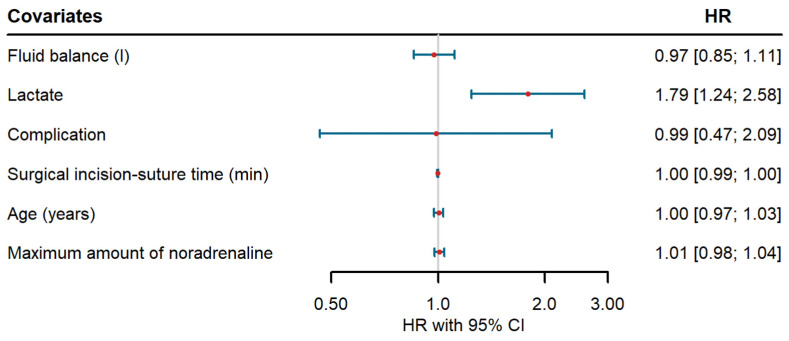
Results of the Cox regression model.

In total, 48 of 73 patients (65.8%) experienced an event of interest within the 5-year follow-up period. Kaplan–Meier analysis unveiled an unadjusted 1-year survival probability of 84.93% (77.11%, 93.55%) and an unadjusted 5-year survival probability of 33.62% (24.3%, 46.53%) in the presented cohort. Cox regression revealed a statistically significant effect of lactate (measured 24 h postoperatively) on the overall 5-year survival (HR 1.79 [1.24, 2.58], Figure 3). A one-unit increase in the lactate level was associated with a 79% elevated risk of dying. There was no statistically significant effect of the fluid balance (HR 0.97 [0.85, 1.11]) or any other considered covariate on the overall 5-year survival (Figure 3).

## 4. Discussion

During the study period, a more liberal volume management approach with an average balance of 9.1 (3.4) l was applied in cytoreductive tumour surgery for ovarian cancer patients at the University Hospital Bonn, Germany. As expected, the fluid balance correlated significantly with the duration of surgery and anaesthesia in this context. Ventilation times were also positively correlated with fluid intake and could, therefore, contribute to a longer ICU stay, which could be avoided or optimised in the future.

Interestingly, it was found that even a generous fluid regime did not lead to less catecholamine administration. In detail, more fluid led to more catecholamine. One of the reasons might be the general hypoalbuminemia in advanced ovarian cancer patients, which may be explained by the large tissue trauma and an early-onset inflammatory response leading to an extravascular fluid loss in the context of capillary leakage and the need for positive inotropic substances, similar to sepsis [13].

Intraoperative fluid substitution is part of the daily routine in anaesthesia, as adequate intravascular volume is essential for sufficient organ perfusion, haemodynamic stability and, thus, for postoperative outcomes [14].

Studies comparing liberal versus restrictive fluid management in terms of goal-directed therapy have partly concluded that the latter offers advantages for the patient [15,16].

On the other hand, there are studies that have shown that restrictive volume management can be associated with a higher rate of acute renal injury [12].

In our study, immediate postoperative renal function, as measured using creatinine levels, showed no impairment in the present patient cohort, suggesting adequate volume therapy. Ultimately, we could not detect a statistically significant impact on long-term survival. It, therefore, remains unclear whether the patients would have benefited from more restrained volume administration.

Our investigation revealed, however, that a liberal approach can have negative consequences, such as increased intraoperative vasopressor consumption, prolonged ventilation times in the intensive care unit, anastomotic insufficiencies and pleural effusions. The observed influence of an increased fluid intake on the overall incidence of postoperative complications has likewise been shown by others. Hasselgren et al. demonstrated major postoperative complications in up to 30% of patients who received at least 3.0 L of fluids during surgery for advanced epithelial ovarian cancer, with an adjusted odds ratio of 33.7 for those with an intake >5000 mL [17]. Therefore, it is desirable to strive for optimal volume management that is beneficial for the patient. Goal-directed therapy aims to optimise the patient’s haemodynamic situation perioperatively, but this may require extended intraoperative monitoring, which may not be universally available [18,19].

Nevertheless, this should be used for high-risk surgical procedures whenever possible.

Since relatively little is known about the factors associated with long-term survival in ovarian cancer patients [20,21], one aim of this study was to investigate whether intraoperative volume management has an influence in this regard.

A meta-analysis by Som et al. concluded that, when considering mortality, goal-directed fluid therapy does not offer a superior benefit compared to the standard treatment [22]. In line with that, our results likewise revealed no significant influence of the fluid balance on long-term survival.

An increased lactate level (24 h following surgery) was the only significant predictor of 5-year survival in the cohort studied. This is in line with the results of Meregalli et al., who found an association between increased lactate levels and higher mortality in high-risk surgical patients [23].

If a perfusion mismatch occurs with a consecutive reduction in the oxygen supply to the tissues, this is reflected in rising lactate values, although other clinical parameters do not always immediately mirror this. In this study, it was shown that, in patients undergoing major cytoreductive surgery, elevated postoperative lactate levels can also be predictive of morbidity, in contrast to all other factors investigated. This could contribute to therapy optimisation, especially if advanced haemodynamic monitoring is available.

### Strength and Limitations

This study had some limitations. First, the patient population was heterogeneous, with varying degrees of organ involvement and, thus, different extents of surgery. Secondly, the decision to substitute fluids was up to the anaesthetist and was not subject to a fixed protocol. Another limiting factor was the non-randomised and retrospective, observational study design. However, performing a post hoc power calculation revealed a statistical power of 86.7% when the overall incidence of postoperative complications in our cohort was compared to previously published values for a similar patient population (from Hasselgren et al.; see above) [17].

Additionally, we were able to follow up with the patients over a long period of time and, thus, record the long-term mortality of a relatively large patient collective. The relationship between pre-existing cardiac conditions and morbidity or mortality was not analysed in this study. However, a follow-up evaluation is already set up, comparing data from extended haemodynamic monitoring with the present cohort to further elucidate this interesting point.

## 5. Conclusions

Volume management in major abdominal surgery remains a particular challenge for the anaesthetist and often follows different hospital standards. The risk of postoperative complications such as pleural effusions or anastomotic insufficiency increases with high fluid administration.

Blood lactate levels appear to be a sensitive marker for predicting an increased mortality risk during major surgery.

However, generous volume administration did not seem to influence the mortality rate and long-term survival in the present patient population.

Generalizability: In order to develop an optimal intraoperative therapy strategy and avoid complications, a corresponding protocol was developed at University Hospital Bonn based on the available study data, which implements extended haemodynamic monitoring and more adjusted volume administration. This could offer advantages in the future for the treatment of the patient clientele studied here.

## Figures and Tables

**Table 1:** Overview of patient characteristics, along with clinical parameters.

	Overall (n = 73)	
Anaesthesia and surgery times		
Surgical incision-to-suture time (min)	Mean (SD)	403 (80)
	Median [IQR]	405 [335, 460]
Anaesthesia time (min)	Mean (SD)	529 (106)
	Median [IQR]	525 [445, 600]
Ventilation time (min)	Mean (SD)	2190 (2710)
	Median [IQR]	1260 [690, 2730]
Laboratory values		
Max. amount of noradrenaline (µg/min)	Mean (SD)	11.6 (12.4)
	Median [IQR]	9 [3, 15]
	Missing	7 (9.6%)
Lactate (mmol/L)	Mean (SD)	1.9 (1.04)
	Median [IQR]	1.7 [1.06, 2.8]
	Missing	9 (12.3%)
Creatinine (day 3) (mg/dL)	Mean (SD)	0.73 (0.29)
	Median [IQR]	0.63 [0.55, 0.83]
	Missing	10 (13.7%)
Complications		
Any complication	yes	34 (46.6%)
	Missing	1 (1.4%)
Pleural effusion	yes	25 (34.2%)
	Missing	6 (8.2%)
Anastomotic insufficiency	yes	7 (9.6%)
	Missing	2 (2.7%)
Ascites	yes	4 (5.5%)
	Missing	4 (5.5%)
Other complications	yes	18 (24.7%)
	Missing	1 (1.4%)

**Table 2:** Overview of composition of fluid intake (L), fluid output (L) and fluid balance (L) in the patient cohort.

	Overall (n = 73)	
Total fluid intake (L)	Mean (SD)	11.2 (4)
	Median [IQR]	11.5 [7.9, 14.1]
Red blood cell concentrate (L)	Mean (SD)	0.73 (0.68)
	Median [IQR]	0.58 [0, 1.16]
Fresh frozen plasma (L)	Mean (SD)	0.69 (0.78)
	Median [IQR]	0.58 [0, 1.16]
Platelet concentrate (L)	Mean (SD)	0.008 (0.05)
	Median [IQR]	0 [0, 0]
Colloids (L)	Mean (SD)	0.62 (0.66)
	Median [IQR]	0.5 [0, 1]
Crystalloids (L)	Mean (SD)	9.1 (3.5)
	Median [IQR]	9 [6.5, 11.5]
Total fluid output (L)	Mean (SD)	2.1 (1.3)
	Median [IQR]	1.8 [1.2, 2.7]
Urine output (L)	Mean (SD)	0.87 (0.67)
	Median [IQR]	0.7 [0.48, 1.05]
	Missing	2 (2.7%)
Blood loss (L)	Mean (SD)	1.3 (0.96)
	Median [IQR]	1 [0.6, 1.6]
Fluid balance (L)	Mean (SD)	9.1 (3.4)
	Median [IQR]	9.5 [6.4, 11.4]

**Table 3:** Spearman’s correlation coefficients (95% confidence interval) between fluid intake (A) (fluid balance (B)) and anaesthesia and surgery times and laboratory measures.

	Fluid Intake	Fluid Balance
	Spearman’s ρ (95%-CI)	Spearman’s ρ (95%-CI)
Surgical incision-to-suture time (min)	0.54	0.48
	(0.36, 0.69)	(0.28, 0.64)
Anaesthesia time (min)	0.59	0.54
	(0.42, 0.73)	(0.35, 0.68)
Ventilation time (min)	0.66	0.69
	(0.50, 0.77)	(0.55, 0.79)
Maximum amount of noradrenaline (µg/min)	0.47	0.52
	(0.26, 0.64)	(0.32, 0.68)
Lactate (mmol/L)	0.33	0.36
	(0.09, 0.53)	(0.12, 0.55)
Creatinine (Day 3) (mg/dL)	0.03	0.06
	(−0.22, 0.28)	(−0.19, 0.31)

## Data Availability

Data are contained within the article.
